# Moringa as a Functional Food for Rheumatoid Arthritis: A Scoping Review of Evidence

**DOI:** 10.3390/biomedicines14030565

**Published:** 2026-03-01

**Authors:** Hiba Murtadha Al-Saadi, Sophia Ogechi Ekeuku, Jasmine Jia Thung Wong, Nurul Nabihah Zahanordin, Norliza Muhammad, Kok-Yong Chin

**Affiliations:** Department of Pharmacology, Faculty of Medicine, Universiti Kebangsaan Malaysia, Cheras 56000, Malaysia; hiba.alsaadi2016@yahoo.com (H.M.A.-S.); virgosapphire2088@yahoo.com (S.O.E.); jasmine3392@gmail.com (J.J.T.W.); nurulnabihahzahanordin@gmail.com (N.N.Z.)

**Keywords:** arthroses, cartilage, inflammation, oxidative stress, phytochemicals

## Abstract

**Background/Objectives**: Rheumatoid arthritis is a chronic autoimmune disease characterised by persistent synovitis and joint destruction. While conventional pharmacotherapies, such as disease-modifying anti-rheumatic drugs, are effective, they are often limited by significant adverse effects and high costs. Moringa, a medicinal plant rich in bioactive compounds, has emerged as a potential functional food adjunct for managing this condition. This scoping review systematically maps the evidence regarding the efficacy of moringa supplementation in alleviating the pathology of rheumatoid arthritis. **Methods**: A comprehensive search of PubMed, Scopus, and Web of Science was performed using a standardised search string to identify original articles investigating the effects of moringa on models of or patients with rheumatoid arthritis. **Results**: A total of 19 eligible studies, comprising in vitro models, preclinical animal studies, and human clinical trials, were included. Phytochemical profiling revealed the presence of potent anti-inflammatory and antioxidant constituents, including flavonoids and isothiocyanates, in various plant parts. Preclinical findings demonstrated that moringa extracts significantly inhibited paw oedema, pannus formation, and cartilage erosion by downregulating proinflammatory cytokines (tumour necrosis factor-alpha, interleukin-1 beta, and interleukin-6) and suppressing nuclear factor kappa B signalling. Clinical trials corroborated these benefits, showing that moringa leaf extracts were associated with reduced disease activity scores and systemic inflammatory markers in patients. Additionally, moringa supplementation alleviated depression associated with rheumatoid arthritis, suggesting a dual therapeutic impact. **Conclusions**: The current evidence supports moringa as a promising functional food adjunct, though further standardised trials are warranted to establish optimal dosing and clinical guidelines.

## 1. Introduction

Rheumatoid arthritis (RA) is a chronic autoimmune joint disease characterised by persistent synovitis, cartilage erosion, and joint destruction [1]. Its characteristics include joint inflammation, immune cell infiltration, synovial swelling, pannus formation, and articular cartilage destruction. These structural changes result in joint stiffness and pain [2]. The prevalence of RA between women and men is estimated to be 3:1. This sex difference is postulated to be driven by the sex hormone profile [3]. Based on the Global Burden of Disease 2021 study, RA incidence increased from 11.66 [95% uncertainty interval (UI): 9.60–13.94] to 13.48 (95% UI: 11.08–16.06) per 100,000 population over 32 years. A parallel increase in RA-related disability-adjusted life years was observed, from 26.37 (95% UI: 18.43–36.99) to 30.71 (95% UI: 20.82–44.08) per 100,000 population [4]. This translates to tremendous healthcare costs and decreased quality of life for patients with RA [5].

The normal synovium contains fibroblast-like synoviocytes (FLSs) and resident macrophages. In RA, the membrane lining is expanded, and FLSs release high levels of RA-related cytokines and chemokines, adhesion molecules, matrix metalloproteinases (MMPs), and tissue inhibitors of MMPs. These factors contribute directly to local cartilage destruction and synovial inflammation [6]. FLSs also form part of the tertiary lymphoid structures that sustain T-cell and B-cell survival and adaptive immune organisation [7]. Overproduction of proinflammatory cytokines, such as tumour necrosis factor-alpha (TNF-α), interleukin (IL)-1, and IL-17, stimulates inflammation and bone and cartilage degradation [8]. Inflammation and oxidative stress are intricately linked. Increased oxidative stress during chronic inflammation leads to connective tissue degradation and joint and periarticular deformities in RA. In the inflamed joints, the infiltrating immune cells, such as neutrophils, lymphocytes, mast cells, and macrophages, can release free radical species that play an essential role in autoimmunity and inflammation [9,10].

The main objectives of RA therapy are to relieve pain, decrease inflammation, and maintain function and articular structures. Most current medications focus on symptomatic relief rather than curing RA [11]. Nonsteroidal anti-inflammatory drugs (NSAIDs) and low-dose glucocorticoids are the first-line treatment to relieve symptoms. However, they are accompanied by side effects, such as gastric ulceration and gastrointestinal bleeding for NSAIDs and increased risk for osteoporosis, diabetes, obesity and immunosuppression [12]. The second-line treatment involves the use of disease-modifying anti-rheumatic drugs (DMARDs), such as methotrexate. They can decrease rheumatoid factor (RF) levels, C-reactive protein (CRP) levels and erythrocyte sedimentation rate. However, DMARDs are associated with hepatotoxicity, pulmonary toxicity and myelosuppression [13]. Biologics against receptors of cytokines, such as TNF-α, IL-6, and IL-1β, have also been used to treat RA [14]. However, they may increase the risk of infection and tuberculosis reactivation, especially in patients with underlying co-morbidities [15]. Given the limitations of each pharmacotherapeutic approach, the search for alternative agents to complement standard therapies continues.

Moringaceae family (or moringa) is a plant genus family with 13 known species, with *Moringa oleifera* (MO) being the most-recognised species [16]. MO is commonly known as drumstick tree and is a staple food in many parts of the world. It is postulated to have originated in the sub-Himalayan regions of northwest India but is now indigenous to many regions of Southeast Asia, Africa, Arabia, the Pacific, the Caribbean Islands, and South America [17]. Moringa is consumed for its nutritional values and medical benefits, such as antioxidant, anti-inflammatory, antimicrobial and anticancer effects [18]. The bioactivity of moringa is attributed to the myriad of minerals, vitamins, phenolic components, isothiocyanates, alkaloids and carotenoids present in various parts of the plant [19]. For example, moringin, a unique isothiocyanate from moringa, suppresses TNF-α signalling by downregulating the expression of TNF-α receptor [20]. Such bioactivities could be harnessed if MO is used as a food adjunct to standard RA management. There are some narrative reviews that discuss the potential of moringa in broader immune disorder management, but they are not focused on RA [21,22,23].

This scoping review summarises the potential benefits of moringa for RA, which were derived from preclinical and clinical studies using a comprehensive and systematic approach. It examines the types of extracts, regimens, RA models, and outcomes tested, thereby providing an understanding of the field and prompting further studies. Ultimately, we hope that the review provides translational insights for the use of moringa as an adjunct to RA management.

## 2. Methods

This scoping review was planned based on the steps designed by Arksey and O’Malley [24] and followed the checklist of the Preferred Reporting Items for Systematic Reviews and Meta-Analyses for Scoping Reviews [25]. The steps taken were research question identification, literature identification, literature selection, data charting and data synthesis. The study protocol was registered in the Open Science Framework (URL: https://osf.io/yuzct/, accessed on 25 February 2026).

### 2.1. Research Question Identification

This current scoping review aims to address the question “What are the effects of moringa supplementation on RA?” The concept of “population, intervention, comparator and outcomes” (PICO) was used to design the research question (Table 1). The PICO framework was designed to be broad to accommodate all study designs in this scoping review.

### 2.2. Literature Identification

An exhaustive literature search was conducted in January 2026 in PubMed, Scopus, and Web of Science using the search string: arthritis AND (rheumatoid OR inflammatory OR autoimmune) AND (moringa OR “drumstick tree” OR “horseradish tree”). During the search, the string was applied to titles and abstracts to avoid nonspecific results. No temporal filters were applied to the search to ensure a comprehensive mapping of the literature. All articles indexed by the databases from the date of inception to the date of the search were included.

All articles with primary data that aimed to investigate the effects of moringa on RA, regardless of study design, were included. During planning, items published in English, Mandarin, Bahasa Malaysia and Bahasa Indonesia were going to be considered. This criterion was not used because all articles were published in English. For animal studies, both autoimmune models, such as collagen and complete Freund’s adjuvant (CFA), and non-specific inflammatory models, such as formaldehyde, were considered. While autoimmune models better mimic the immunological and pathological changes seen in human RA [26], non-specific inflammatory models are valuable for evaluating the therapeutic effects of moringa in suppressing the acute inflammatory cascades and oxidative stress that lead to joint swelling and pain

Articles without primary data, such as all types of reviews, letters, editorials and perspectives, were excluded. Conference abstracts and proceedings were excluded due to incomplete data and duplication with full articles. 

### 2.3. Literature Selection

The search results from the three databases were combined using EndNote 2025 (version 22.0, Clarivate, London, UK). Automatic deduplication by EndNote was used to remove overlapping articles, followed by manual checking. A list of unique articles was presented to two researchers (H.M.A.S. and S.O.E.) for title and abstract screening based on the inclusion and exclusion criteria. Then, full texts of the eligible articles were retrieved and screened by the same researchers. Since all articles were available online, the authors were not contacted. The reference lists of the included articles were screened to identify any articles missed during the electronic search. Discrepancies among researchers regarding the inclusion of articles were resolved through discussions with the corresponding authors (N.M. and K.Y.C.).

### 2.4. Data Charting

Data from the included articles were extracted by two researchers (H.M.A.S. and S.O.E.) using a standardised Excel sheet (Microsoft, Redmond, WA, USA). The extracted data included authors, year of publication, characteristics of preclinical RA models or patients with RA, treatment regimen (type of extract, dose and treatment period of moringa) and major findings related to RA disease progression. Two other researchers verified the extracted data (J.J.T.W. and N.N.Z.).

### 2.5. Data Synthesis

The data were summarised and reported qualitatively based on the study characteristics and markers of RA disease progression, including joint swelling, pain, inflammation, cartilage and bone structures, joint remodelling markers, and signalling markers. The qualitative synthesis approach was selected due to the wide range of study designs, differences in treatments (plant parts, solvents, doses, and formulations), and different reported outcomes, which substantially limited cross-study comparisons.

## 3. Results

### 3.1. Search Results

**Figure 1:**
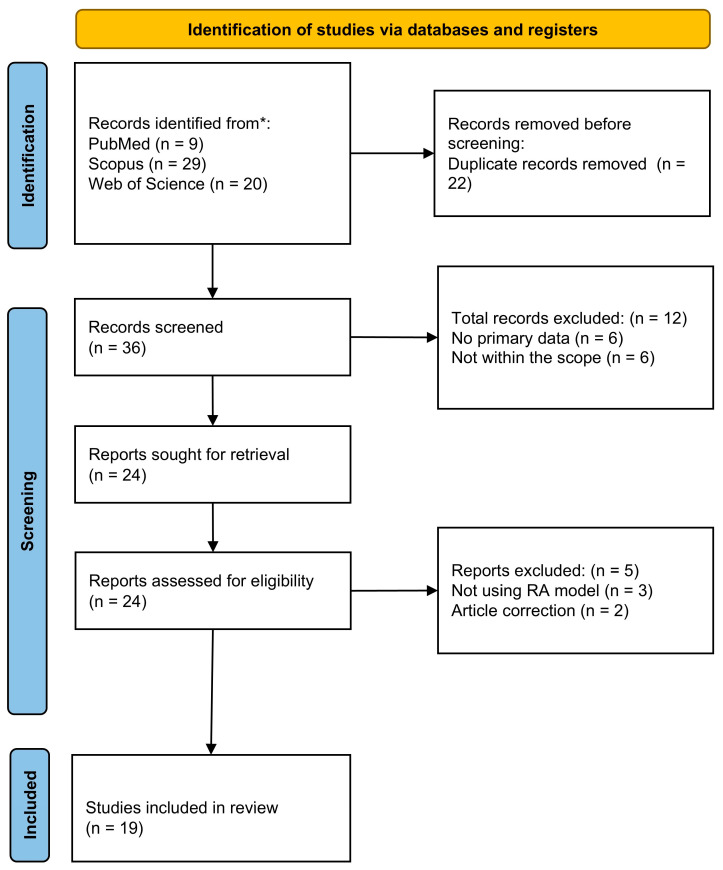
PRISMA-ScR flow chart of the article selection process of this review. * Only online scholarly databases were searched.

The literature search identified 36 unique articles across the three databases. After removing six articles without primary data, two correction articles, six articles with a different objective, and three articles not using RA models, 19 articles were included in the scoping review. The literature search process is summarised in Figure 1.

### 3.2. Study Design

Among the 19 studies, one study utilised primary human FLSs from a 47-year-old RA patient to identify proteomic changes after exposure to MO extract [27]. A total of 13 studies utilised Wistar or Sprague Dawley rats with RA induced using CFA, formaldehyde, turpentine oil, or collagen (collagen-induced arthritis (CIA)) [28,29,30,31,32,33,34,35,36,37,38,39,40]. Five human clinical trials were conducted in a single institution in Indonesia to evaluate MO leaf extracts as adjuvants for RA and its associated psychological morbidity [41,42,43,44,45]. All studies examined extracts from MO species, except Shamlan et al. [40], who examined an extract from *M. peregrina*, and Mukherjee et al. [35], who examined an extract from *M. concanensis*. Among all the studies, only one used a standardised extract [28], which is a preparation that has been processed to contain a certain predetermined amount of marker compound [46]. In this study, granules from a 95% ethanol extract of MO leaves, standardised based on three bioactive phytochemical markers (cryptochlorogenic acid, isoquercetin, and astragalin), were used [28].

### 3.3. Phytochemical Profiling of the Moringa Extract

Phytochemical profiling of *Moringa* species demonstrated that the bioactive constituents vary significantly depending on the plant part (leaves, seeds, flowers, roots, or stem bark) and extraction solvent (Table 2). The studies typically employed a multi-step approach, beginning with preliminary phytochemical screening to identify broad classes such as alkaloids, flavonoids, and tannins, followed by advanced techniques such as high-performance liquid chromatography (HPLC) and gas chromatography–mass spectrometry (GC-MS) for precise quantification and identification of specific compounds. This pipeline is in line with standard practices [47]. The profiling showed that polar extracts (ethanol, methanol, and water) were generally rich in antioxidant polyphenols such as quercetin and kaempferol, while non-polar extracts (hexane) and seed oils were dominated by essential fatty acids and vitamins, such as α-tocopherol.

### 3.4. Treatment Regimen

In an in vitro study, an ethanolic extract of whole moringa leaves (500 μg/mL to 100 mg/mL) was screened for toxicity, and 75 mg/mL was determined to be the optimal non-cytotoxic concentration for human FLSs for a 24 h exposure [27].

Various types of moringa extracts from different parts of the plants have been tested in animal models, including ethanolic (30–600 mg/kg) [32,34], ethyl acetate [37], methanolic (150–600 mg/kg) [33,36,37,38], hexane (30–300 mg/kg) [34] and aqueous extracts of the leaves (150–600 mg/kg) [36,38,39], and formulated granules (95% ethanolic extract with Arabic gum and Tween 20, 250 mg/kg) [28]. Methanolic and hydro-alcoholic root extracts of *M. concanensis* have also been tested (200–400 mg/kg) [33,35]. Individual studies also tested an ethanolic seed kernel extract (100–200 mg/kg) [30], hydro-alcoholic flower extract (100–200 mg/kg) [31] and methanolic stem bark extract (125–500 mg/kg) [29]. One study tested *M. peregrina* leaf (0.5 g/rat) and seed oil extracts (1 mL/rat) individually or in combination [40]. The treatment period lasted 5–10 days for acute models, and 21–30 days for chronic CFA/CIA models.

The moringa extracts tested in clinical trials were leaf extracts packaged as 500 mg capsules. One study utilised a water-macerated extract that was then evaporated to dryness [43]. The dose tested was 2000 mg/day [41,42,45]. Two studies tested a dose of 40.50 mg/kg body weight per day [43,44]. Most human trials were conducted over 28 or 30 days.

### 3.5. In Vitro Evidence

An in vitro study by [27] utilised a proteomic approach to identify the molecular pathways through which moringa restores cellular homeostasis in arthritic synoviocytes. An ethanolic leaf extract (75 mg/mL) significantly altered the abundance of 40 aberrantly expressed proteins in human FLSs. Notably, pathways related to inflammation and aberrant cell proliferation (involving 35 proteins) were suppressed, specifically via the downregulation of Nedd-8 (which facilitates NF-κB transport) and leucine-rich PPR motif-containing protein (which prevents cell apoptosis). This observation was substantiated by the downregulation of NF-κB p65 observed using confocal imaging. Concurrently, several protective proteins were upregulated by the extract, most notably kallistatin (an anti-inflammatory and anti-angiogenic agent) and heat shock 70 kDa protein 8 (which protects the proteasome from stressors) [27].

### 3.6. In Vivo Evidence

#### 3.6.1. Paw Oedema

Preclinical animal models have demonstrated the capacity of various *Moringa* species and plant parts to repair structural damage and normalise systemic biomarkers in arthritis. For instance, ethanolic and methanolic leaf extracts significantly inhibited paw oedema in carrageenan, formaldehyde, and CFA models [32,38]. Notably, standardised ethanol leaf granules (250 mg/kg) demonstrated superior efficacy compared to crude leaf extracts, an effect attributed to improved solubility [28]. Furthermore, ethanolic seed kernel extracts (100–200 mg/kg) significantly reduced both primary lesions (initial swelling) and secondary lesions (systemic inflammation) in CFA-induced arthritis [30]. Similarly, hydro-alcoholic flower extracts significantly reduced the arthritic index and paw volume [31]. In turpentine oil- and formaldehyde-induced models, methanolic stem bark extracts inhibited the global oedematous response, demonstrating efficacies comparable to those of standard drugs, such as aspirin [29].

#### 3.6.2. Joint Function and General Physical Status

Moringa extracts helped maintain normal movement patterns by alleviating pain and joint stiffness. Standardised ethanol leaf granules (250 mg/kg) prevented the disruption of locomotion and gait. The treated rats exhibited a functional index (FI) (within the normal range +11%), whereas untreated arthritic controls displayed significantly impaired movement [28]. Similarly, an ethanolic leaf extract (250 and 500 mg/kg) successfully preserved gait behaviour, maintaining the FI in Sprague Dawley rats throughout a 21-day study [32]. Additionally, methanolic leaf and root extracts (200, 300, and 400 mg/kg) improved rats’ physical capacity by significantly reducing thermal hyperalgesia and mechanical allodynia. This analgesic effect enabled rats to move freely, mitigating the physical limitations typically imposed by chronic pain [33].

Arthritis is frequently associated with physical deterioration and weight loss stemming from limited mobility and systemic inflammation. However, moringa extracts were reported to reverse this trend. Rats treated with an aqueous leaf extract (500 mg/kg) exhibited significantly higher body weight gain compared to untreated arthritic rats, indicating improved overall health and nutritional absorption [39]. A methanolic stem bark extract (125, 250, and 500 mg/kg) induced a dose-dependent increase in body weight [29]. Consistent findings were observed with an ethanolic seed kernel extract (100 and 200 mg/kg) [30], hydro-alcoholic flower extract (100 and 200 mg/kg) [31], and hydro-alcoholic *M. concanensis* root extract (200 and 400 mg/kg) [35], all of which promoted body weight recovery in RA models.

#### 3.6.3. Structural and Cartilage Markers

Moringa extracts have demonstrated a significant capacity to reverse histological damage and preserve the articular cartilage layer. Administration of an aqueous leaf extract (500 mg/kg) significantly reversed the depth of articular cartilage damage in formaldehyde-induced models [39]. Notably, this extract was found to be comparable with vitamin D in repairing histomorphological alterations of the cartilage [39]. In other studies, treatment with methanolic and aqueous leaf extracts (600 mg/kg) or ethanolic seed extracts (200 mg/kg) successfully prevented cartilage erosion [30,37].

#### 3.6.4. Subchondral Bone

Moringa extracts were reported to protect underlying bone structure by preventing erosion, promoting mineral density, and inhibiting pannus formation. Radiographic assessments confirmed that standardised ethanol leaf granules (250 mg/kg) and ethanol leaf extracts (250–500 mg/kg) significantly reduced subchondral erosion and osteoporosis [28,32]. These treatments helped maintain the integrity of the joint space, which otherwise narrows due to bone loss [28,32]. A hallmark of joint destruction is the formation of pannus, an abnormal fibrovascular tissue that erodes into the bone; high doses of methanolic and aqueous leaf extracts (600 mg/kg) were shown to prevent pannus formation, thereby halting bone destruction at the cartilage–bone interface [38].

#### 3.6.5. Inflammatory Markers

Various moringa extracts were reported to suppress inflammation in arthritic models. Administration of an ethanolic seed kernel extract (100–200 mg/kg), methanolic leaf extract (600 mg/kg), or aqueous leaf extract (200 mg/mL) significantly decreased serum RF and CRP levels [30,36,38]. Additionally, a hydro-alcoholic flower extract (200 mg/kg) and methanolic leaf extract (600 mg/kg) significantly downregulated mRNA expression and serum levels of TNF-α, IL-1β, and IL-6 [31,38]. Conversely, *M. peregrina* seed oil (1 mL) and a methanolic leaf extract (150–600 mg/kg) significantly upregulated the circulating anti-inflammatory cytokines IL-4 and IL-10 [38,40]. Furthermore, a methanolic leaf extract (600 mg/kg) significantly reduced cyclooxygenase-2 expression and prostaglandin E_2_ concentrations [38].

#### 3.6.6. Redox Markers

Moringa extracts also improved antioxidant defence in rat models, but these parameters were not measured in the joint. An ethanolic seed extract (200 mg/kg) and methanolic leaf extract (600 mg/kg) reinstated SOD and CAT activities while significantly reducing the levels of the lipid peroxidation product malondialdehyde (MDA) in the liver [30,37].

### 3.7. Clinical Evidence

Administration of a moringa leaf extract showed preliminary efficacy in modulating disease activity and inflammatory markers in specific RA patient cohorts. High-dose regimens (1000 mg twice daily) or weight-based dosing (40–50 mg/kg/day) were associated with a reduction in DAS28-hsCRP and Simplified Disease Activity Index (SDAI) scores within the study period [43,44]. Mechanistically, this appears to correlate with a significant downregulation of acute-phase reactants and cytokines. Treatment was reported to decrease the levels of serum amyloid A (SAA), a reactant that is highly sensitive to active synovitis, and reduce serum IL-6 levels [41,43]. Additionally, daily intake of 2000 mg was found to improve certain haematological markers of systemic inflammation, evidenced by reductions in the neutrophil-to-lymphocyte ratio and mean platelet volume [42].

In addition to its observed anti-inflammatory properties, the moringa leaf extract demonstrated potential supportive benefits for RA-associated comorbidities in preliminary trials. In patients presenting with comorbid depression, a lower dosage of 500 mg twice daily was associated with reduced depression severity, as measured by BDI-II scores. This psychological improvement was accompanied by a reduction in serum cortisol levels, suggesting a potential regulatory effect on the hypothalamic–pituitary–adrenal axis [45]. This observation requires further validation in larger studies.

### 3.8. Comparison with Standard RA Therapies

In various preclinical models, moringa extracts demonstrated potential anti-inflammatory and anti-arthritic activities that warrant further investigation alongside conventional controls such as NSAIDs, dexamethasone, methotrexate, and vitamin D.

In a rat RA model, a high dose of a methanolic stem bark extract (500 mg/kg) demonstrated inhibitory effects on turpentine oil-induced swelling, similar to those of aspirin (100 mg/kg). Furthermore, moringa at this dosage exhibited a notable reduction in the global oedematous response in proliferative models [29].

Moringa leaf extracts and granules show potent anti-inflammatory and analgesic effects comparable to indomethacin. In chronic arthritis rat models, a moringa leaf extract (250 mg/kg) demonstrated comparable oedema inhibition in the chronic phase, approaching the levels of indomethacin [32]. While indomethacin typically showed a faster onset of action, moringa’s ability to maintain a normal FI and reduce thermal hyperalgesia was noted to be similar to indomethacin [28,32,33].

A methanolic leaf extract (600 mg/kg) showed a maximum percentage inhibition of paw volume that was comparable to piroxicam at the end of a 10-day animal study [37]. The protective effect of both aqueous and methanolic extracts on pannus formation and bone erosion was found to be comparable to piroxicam in the laboratory setting [38].

Compared with dexamethasone, moringa exhibited similar effects on systemic cytokines and joint histology. Ethanolic seed extracts (200 mg/kg) were reported to reduce serum levels of TNF-α and IL-1 and normalise MDA levels in a manner similar to dexamethasone [30]. Histopathological observations showed that both a moringa flower extract (200 mg/kg) and dexamethasone provide significant protection against bone destruction and cartilage erosion in rats with RA [31].

Compared with vitamin D, moringa was reported to be a potentially strong agent for structural cartilage repair in rats. An aqueous leaf extract (500 mg/kg) was found to be effective in repairing histomorphological alterations and reverting the depth of articular cartilage damage compared to vitamin D (4000 IU/kg) in a rat model of arthritis [39]. Additionally, moringa increased body weight more markedly than vitamin D, likely due to its unique combination of anti-inflammatory and high nutritional properties [39].

In a rat study, a hydro-alcoholic root extract of *M. concanensis* (200–400 mg/kg) exhibited anti-arthritic effects comparable to those of methotrexate (0.75 mg/kg) in reducing physical and biochemical indicators of RA [35].

A noteworthy observation across studies was that moringa exhibited a strong safety profile compared to positive controls within the reported range. Rats treated with moringa showed better weight recovery compared to the indomethacin-treated group [28,32]. Furthermore, while methotrexate is often associated with liver damage, moringa extracts did not appear to cause significant histopathological alterations in the liver within the tested range [35,37].

A summary of the effects of moringa on RA is presented in Table 3.

## 4. Discussion

**Figure 2:**
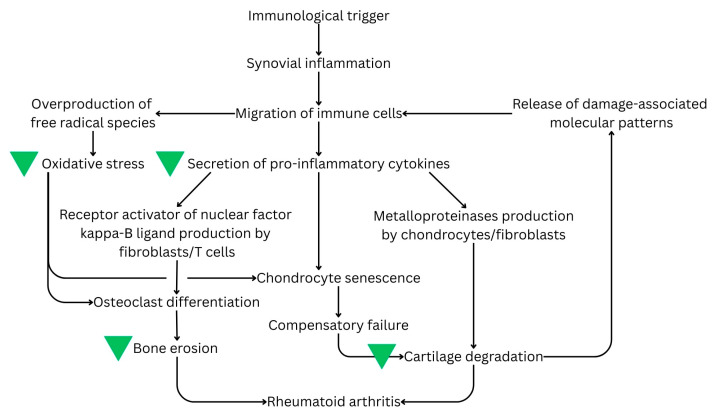
Integrated effects of moringa on RA pathology. The green triangle signifies the hierarchical impact of moringa supplementation, ranging from reducing inflammatory and oxidative stress to localised structural preservation of the joint.

Mechanistically, the evidence for moringa’s efficacy can be divided into direct, localised effects and indirect, systemic outcomes. Direct findings are evidenced by histopathological analysis of joint tissues, demonstrating reduced pannus formation, cartilage erosion, preserved subchondral bone, and localised downregulation of MMPs within the synovium. In contrast, indirect findings include the modulation of systemic inflammatory markers, such as serum CRP, IL-6, and ESR, as well as antioxidant parameters measured at the liver. While these systemic changes provide evidence of a reduced inflammatory state, they serve as a proxy for, rather than direct evidence of, intra-articular structural preservation. The general mechanism of moringa’s anti-RA action is presented in Figure 2.

As with other natural products, there are challenges in translating moringa’s anti-RA potential from preclinical models to clinical practice. The foremost challenge is the pharmacokinetics of moringa. Many animal studies included in this review utilised moringa leaf extracts at dosages of 200–500 mg/kg body weight/day in rat models of RA. Translating this dosage range to human equivalent doses (assuming a 70 kg human) using body surface ratio scaling gives a range of 2.3–5.7 g/day [48]. Based on the reported yield of the water maceration technique (12–15%), this would require a daily consumption of 15–20 g of raw moringa leaves [49]. This amount may pose significant compliance challenges due to the leaves’ palatability and gastrointestinal burden. Thus, the extraction of bioactive compounds from moringa would be necessary for supplementation. As demonstrated by a series of clinical studies included in this review, 2 g/day of a moringa leaf extract was administered to patients with RA, and some positive results were observed [41,42,43,44,45].

Furthermore, the clinical utility of moringa in RA is heavily dependent on the systemic bioavailability of its bioactive isothiocyanates, particularly glucomoringin (GMG) and moringin. An in silico pharmacokinetic profiling study suggested low gastrointestinal absorption of GMG (~4%) [50]. Currently, in vivo pharmacokinetic studies on moringa are scarce. A notable study in rats identified 42 compounds following MO leaf supplementation (30 g/kg after an overnight fast), and pharmacokinetic profiles of eight compounds (chlorogenic acid, cryptochlorogenic acid, vicenin-2, vitexin, rutin, isoquercitrin, kaempferol-3-O-rutinoside, and astragalin) are available [51]. They could serve as biomarkers of MO absorption in future studies. The use of advanced encapsulation materials could potentially enhance the oral bioavailability of MO’s bioactives [52], allowing for more manageable clinical doses to be administered to patients with RA while maintaining therapeutic concentrations at target organs, such as the joints.

Another challenge to translating moringa into clinical use is the pervasive lack of standardisation of extraction across studies, which often renders results incomparable and irreproducible in a clinical setting. The phytochemical profile of moringa extracts could depend on geographical, climate and extraction methods [53,54]. Future clinical trials should abandon simple weight-based dosing (e.g., “500 mg capsule”) in favour of biomarker-standardised formulations. The biomarkers should be specific to moringa and exhibit potent bioactivity, such as GMG or moringin. By adopting good manufacturing practice standards that guarantee a minimum concentration of these specific bioactive markers, researchers can ensure that negative trial results are due to a lack of efficacy rather than underdosing of the active components of the moringa extracts. From an agricultural perspective, standardising planting conditions and housing moringa in a controlled environment, such as greenhouses, and introducing quality controls in planting practices may also improve the consistency of moringa product quality. 

The clinical translation of moringa should also address the realistic aspect of polypharmacy in patients with RA. The standard of care for RA often involves a complex regimen of agents, including corticosteroids, and conventional and synthetic DMARDs. This creates a precarious environment for potential herb–drug interactions (HDIs). The available literature suggests a protective effect of moringa on methotrexate-induced organ toxicity, especially in the liver, through the regulation of redox status and apoptosis [55,56,57,58]. These studies position moringa as a suitable adjuvant to methotrexate therapy in RA that prevents the side effects of the drug. However, no other interaction study between moringa and other RA therapies is available.

An in vitro study investigating HDIs indicated that MO extracts may inhibit key cytochrome P450 enzymes, including CYP3A4, CYP1A2, and CYP2D6. These effects were specifically attributed to the plant’s flavonoid and isothiocyanate components [59]. This is clinically significant because many anti-RA medications (e.g., tofacitinib, corticosteroids) are substrates of these enzymes [60,61]. If MO inhibits CYP3A4, it could theoretically decrease the clearance of these drugs, leading to supratherapeutic serum levels and toxicity. Conversely, if MO induces P-glycoprotein efflux transporters, it might reduce intracellular drug concentrations, making therapy ineffective. A recent study involving nevirapine (a CYP3A4 substrate) showed no significant alteration in pharmacokinetics with concurrent MO use in adults with HIV [62], but similar findings on RA drugs with a narrow therapeutic index are not available. This warrants in-depth interaction studies on MO and other RA agents to ensure it does not significantly alter the drugs’ pharmacokinetics.

Overall, moringa should be positioned to complement the current RA therapies, not replace them, especially among patients experiencing flares. To validate the adjunct role of moringa, researchers should design a randomised controlled trial (RCT) focusing on RA patients with RA who have achieved inflammatory remission but still suffer from oxidative-stress-driven symptoms like fatigue and cognitive dysfunction. When comparing standard therapy plus moringa against standard therapy plus a placebo, researchers should utilise primary endpoints that extend beyond the DAS28 score to ensure a more comprehensive assessment. They should incorporate patient-reported outcomes, such as the FATIGUE-VAS, and biological markers of oxidative damage (e.g., lipid hydroperoxides or F2-isoprostanes), rather than relying solely on erythrocyte sedimentation rates. This approach isolates the specific mechanistic contribution of moringa, i.e., restoring redox balance and improving quality of life, rather than expecting it to compete directly with immunosuppressants in halting bone erosion. By narrowing the inclusion criteria to this stratified group, the statistical power to detect a meaningful clinical benefit increases significantly.

The quality of the studies included in this review varies. Most preclinical animal studies employed standard models (CIA and CFA), yet many lacked explicit reporting on randomisation and blinding, which are critical for minimising performance bias. Regarding the clinical evidence, most trials were single-centre studies with a limited number of patients, which may limit the generalisability of the findings. Furthermore, the lack of a placebo-controlled, double-blind design in the human studies introduces the potential for observer bias in subjective outcomes such as BDI-II scores or pain scores. Future research should prioritise multi-centre, double-blind RCTs to strengthen the evidence base for moringa in RA management.

Several limitations in the current literature temper our confidence in drawing definitive clinical conclusions. First, the predominance of preclinical studies over human clinical trials means that the translatability of moringa’s anti-RA efficacy to diverse human populations remains uncertain. Second, the lack of standardised extraction protocols across studies introduces significant variability in phytochemicals. This inconsistency limits the ability of this review to recommend specific therapeutic doses or identify the most potent moringa species. Furthermore, all the clinical trials had small sample sizes and short follow-up periods, which restricts the confidence in the long-term safety and sustained efficacy of moringa as a chronic functional food adjunct. Collectively, these factors indicate that while the preliminary evidence is promising, it is insufficient to support its use as a primary treatment in clinical guidelines at this stage.

This review is not without its limitations. We only searched three databases and did not search for grey literature, so selection bias cannot be ruled out. We also did not conduct a quantitative synthesis of the data due to disparate study designs, types and doses of the extract used, and reported outcomes. Hence, the overall effect size of the treatment cannot be deduced. The limited clinical data (all from one institution) and the absence of a long-term study prevent generalizing the findings to more heterogeneous populations and patients with chronic conditions. An additional search of clinicaltrials.gov on 24th February 2026 confirmed the absence of ongoing moringa trials on RA.

On the other hand, the review provides a robust translational synthesis by rigorously mapping evidence from molecular mechanisms to clinical outcomes, laying the groundwork for future research to verify moringa’s role as an adjunct treatment for RA. Although this review includes studies dating back to 2007, the primary disease models employed (e.g., CFA-induced arthritis) remain the gold standard in preclinical RA research. Furthermore, the foundational phytochemical screenings in these earlier studies provided the basis for more advanced compositional characterisations, such as HPLC and GC-MS, which have since confirmed the presence of the same key bioactive isothiocyanates and flavonoids.

## 5. Conclusions

The current literature indicates that moringa is a potential functional food adjunct in the management of RA. Preclinical data demonstrate its capacity to intercept the disease process at both the inflammatory and oxidative checkpoints, effectively mitigating synovial pannus formation and cartilage erosion. Preliminary clinical trials corroborate these findings, showing significant reductions in disease activity scores and inflammatory markers. Moringa also shows the ability to simultaneously address systemic comorbidities such as depression and metabolic dysregulation.

However, the transition from a promising candidate to a clinical standard of care is currently impeded by the lack of phytopharmaceutical standardisation and rigorous pharmacokinetic data. The heterogeneity of extract preparations and the paucity of long-term safety profiles regarding HDIs remain significant barriers. Therefore, future research must prioritise standardised, biomarker-quantified clinical trials to establish precise dosing guidelines. Until then, MO represents a promising, accessible and supportive therapy for managing residual symptoms and improving quality of life in patients with RA.

## Figures and Tables

**Table 1:** PICO framework for the scoping review.

Item	Explanation
Population	In vitro or in vivo models of RA, patients with RA
Intervention	Moringa extract, any solvent
Comparator	Untreated cells, animals or patients with RA
Outcomes	All health outcomes related to RA disease progression

**Table 2:** Studies that performed phytochemical profiling of moringa.

Study	Extract Type	Profiling Method	Identified Constituents
[30]	Ethanolic (seeds)	Preliminary screening; isolation	Alkaloids, flavonoids, saponins, glycosides, steroids, tannins, terpenoids; benzylisothiocyanate, moringine, niazimicin, niazirin, β-sitosterol.
[31]	Hydroalcoholic (flowers)	Preliminary screening	Flavonoids, polyphenols, tannins, triterpenoids; rhamnetin, kaempferol, quercetin, vitamins A, B, C.
[33]	Methanolic (leaf/root)	Preliminary screening; HPLC	Saponins, terpenoids; quercetin.
[29]	Methanolic (stem bark)	Preliminary screening	Tannins, flavonoids, anthraquinones, saponins.
[34]	Hexane & ethanol (leaves)	UHPLC; mass spectroscopy (negative-ion mode)	Ethanol: Kaempferol-3-glucoside. Hexane: Fatty acids (palmitic, arachidic, behenic, chlorogenic, lignoceric, eicosatetranoic).
[37]	Methanolic, hexane, ethyl acetate, butanol, aqueous (leaves)	Qualitative tests; HPLC	Kaempferol, quercetin, gallic acid, vanillic acid, caffeic acid, p-coumaric acid, sinapic acid, ferulic acid.
[38]	Methanolic & aqueous (leaves)	GC-MS; HPLC	Hexadecanoic acid methyl ester, 10-octadecenoic acid, phytol, vitamin E (α-tocopherol), quercetin, kaempferol.
[28]	95% Ethanol (leaf granules)	HPLC-UV	Cryptochlorogenic acid, isoquercetin, astragalin (kaempferol-3-O-glucoside).
[35]	Hydro-alcoholic and methanol extracts (*M. concanensis* root)	Preliminary screening	Alkaloids, flavonoids, phenols, terpenoids, tannins, steroids (methanol extract only), carbohydrates.

**Table 3:** Detailed summary of moringa studies in rheumatoid arthritis.

Study	Basic Characteristics (Sex, Age, Weight)	RA Induction/Severity	Moringa Treatment, Dose & Period	Grouping (N per Group & Standard Therapy)	Parameters That Decreased Significantly (vs. RA Control)	Parameters That Increased Significantly (vs. RA Control)	Parameters That Were Unchanged (vs. RA Control)
[28]	Male Sprague Dawley rats; 8–10 weeks old; 150–180 g	CFA (0.1 mL sub-plantar)	Ethanol extract (EE)/granule formulation from MO leaves, 250 mg/kg, po, 21 days	RA Control: *n* = 6; Moringa: *n* = 6 for EE, 6 for G; Positive Control: *n* = 6 (indomethacin, 2.5 mg/kg)	EE & G: Paw thickness, arthritic score & ESR	EE & G: Hb, RBCs & PCV EE: Body weight.	EE & G: WBCs
[29]	Male and female Wistar rats; age not specified; 150–200 g	Turpentine/formaldehyde/CFA (doses not specified)	Methanolic extract of stem bark of MO, 125–500 mg/kg, po, 10 or 21 days	RA Control: *n* = 6; Moringa: *n* = 6; Positive Control: *n* = 6 (aspirin, 100 mg/kg)	Paw oedema, arthritic index, ESR (125 mg/kg only) & WBCs	Body weight, Hb & RBCs	Not available
[30]	Female Wistar rats; age not specified; 150–180 g	CFA (0.1 mL, 10 mg/mL *M. tuberculosis*)	Ethanolic seed extract, 100 or 200 mg/kg, po, 21 days	RA Control: *n* = 6; Moringa: *n* = 6/dose; Positive Control: *n* = 6 (dexamethasone, 5 mg/kg)	Paw oedema, arthritic index, RF, TNF-α, IL-1, IL-6, SOD & CAT	MDA & body weight	GSH
[32]	Male Sprague Dawley rats; 8–10 weeks old; 150–200 g	CFA (0.1 mL, 10 mg/mL *M. tuberculosis*)	Ethanol leaf extract, 250 or 500 mg/kg, po, 21 days	RA Control: *n* = 6; Moringa: *n* = 6/dose; Positive Control: *n* = 6 (indomethacin, 2.5 mg/kg)	Paw oedema, arthritic index, ESR, hyperalgesia & radiographic score	Body weight, Hb, RBCs (250 mg/kg only), PCV & WBCs (500 mg/kg only)	Not available
[31]	Male and female Wistar rats; 9–10 weeks old; 150–180 g	CFA (0.1 mL sub-plantar)	Hydroalcoholic extract of MO flowers, 100 or 200 mg/kg, po, 21 days	RA Control: *n* = 6; Moringa: *n* = 6/dose; Positive Control: *n* = 6 (dexamethasone, 2.5 mg/kg)	Paw volume, arthritic index (200 mg/kg only), RF, TNF-α, IL-1 & ESR.	Body weight	Not available
[35]	Female Wistar rats; age not specified; 150–200 g	CFA (0.1 mL sub-plantar)	Hydro-alcoholic root extract (*M. concanensis*), 200/400 mg/kg, po, 21 days	RA Control: *n* = 6; Moringa: *n* = 6/dose; Positive Control: *n* = 6 (methotrexate, 0.75 mg/kg)	Paw volume (trend), joint diameter (trend), CRP, RF, ALP, ALT & total cholesterol	Not available	Not available
[38]	Both sexes Wistar rats; age not specified; 170–220 g	CFA (150 mL emulsion)	Methanolic/aqueous leaf extract, 150–600 mg/kg, po, 21 days	RA Control: *n* = 6; Moringa: *n* = 6; Positive Control: *n* = 6 (piroxicam, 10 mg/kg)	RF, CRP, PGE2, TNF-α, COX-2, IL-1β, IL-6, NF-κB, MDA & immune organ weight	I-κB, IL-4, IL-10, CAT, SOD, Hb, RBCs & body weight	AST, ALT, urea & creatinine
[37]	Male and female Wistar rats; age not specified; 150–200 g	Formaldehyde (0.1 mL 5%)	Ethyl acetate/methanolic/aqueous MO leaf extract, 150–600 mg/kg, po, 10 days	RA Control: *n* = 6; Moringa: *n* = 6/dose; Positive Control: *n* = 6 (piroxicam, 10 mg/kg)	Paw volume/diameter, pannus formation, bone erosion, TLC & platelets	SOD, CAT, RBCs, Hb & body weight	Bilirubin, ALT & AST
[39]	Male Sprague Dawley rats; 2 months old; 300 g	Formaldehyde (0.1 mL 2% v/v)	Aqueous MO leaf extract, 500 mg/kg, po, 28 days	RA Control: *n* = 10; Moringa: *n* = 10; Positive Control: *n* = 10 (vitamin D, 4000 IU/kg)	Articular cartilage damage depth	Body weight	Not available
[40]	Male Wistar rats; 5–6 weeks old; 160–190 g	CFA (0.1 mL, 10 mg/mL *M. tuberculosis*)	Dried leaves (500 mg) and/or seed oil of *M. peregrina* (1 mL), 30 days	RA Control: *n* = 5; Moringa: *n* = 5/treatment; Positive Control: not available	RF, IL-6, IL-1β, TNF-α & growth indicators	IL-4, IL-10 & body weight	IL-1α, IL-12p70, IL-17A, IL-13 & food intake
[34]	Male & female Wistar rats; age not specified; 180–200 g	Formalin/collagen-II	Hexane/ethanol extract of leaves of MO, 30–300 mg/kg, po, 35 days	RA Control: *n* = 6; Moringa: *n* = 6/extract; Positive Control: *n* = 6 (naproxen 10 mg/kg or ketorolac 0.58 mg/kg or dexamethasone 0.1 mg/kg)	Flinching behaviour, paw oedema & mechanical hyperalgesia	Not available	Not available
[36]	Male Wistar rats; 4–9 months old; 350–450 g	Formaldehyde (0.1 mL 2% conc.)	Aqueous MO leaf extract (concentration: 200 mg/mL), 1.5 mL/rat, po, 4 doses over 30 days (or/and low-level laser therapy)	RA Control: *n* = 10; Moringa: *n* = 10; Positive Control: *n* = 10 (low-level laser therapy)	RF, WBCs, ESR, cholesterol, LDL, TG & VLDL	HDL	Not available
[33]	Male Wistar rats; age not specified; 200–220 g	CFA (10 mg/mL in 0.1 mL mineral oil)	Methanolic MO leaf/root extract, 200–400 mg/kg individually or 200 mg/kg combined, po, 3 doses	RA Control: *n* = 6; Moringa: *n* = 6/extract/dose; Positive Control: *n* = 6 (indomethacin, 5 mg/kg)	Doses > 200 mg/kg or combined extract: thermal hyperalgesia & mechanical allodynia	Not available	Not available
[43]	Human females; mean age: 18–60 years	SDAI score: ~24.5 (active RA)	MO leaf extract, 40.50 mg/kg body weight/day, po, 1 month	Moringa Group: *n* = 15; Placebo Group: *n* = 15	IL-6 levels & SDAI score	Not available	Not available
[44]	Mostly female patients; mean age: 42	DAS28-hsCRP score: ~4.5	MO leaf extract (Keloreena^®^), 1000 mg twice daily, po, 30 days	Moringa Group: *n* = 15; Placebo Group: *n* = 15	DAS28-hsCRP score	Not available	Not available
[41]	Mostly female patients; median age: 49.50	Elevated APRs (SAA, hs-CRP, ESR)	MO leaf extract, 1000 mg twice daily, po, 28 days	Moringa Group: *n* = 20; RA Control Group: *n* = 20 (standard therapy only)	Serum amyloid A (SAA)	Not available	Not available
[45]	Mostly female patients (56.25%); age: 21–60	BDI-II score > 20 (depressed RA)	MO leaf extract, 2 × 500 mg twice daily, po, 28 days	Moringa Group: *n* = 16; Placebo Group: *n* = 16	BDI-II (depression) score, serum cortisol & WBCs	Not specified	RF & CRP
[42]	Autoimmune patients; Age/sex/weight not available in source	Active inflammation	MO leaf extract, 2000 mg/day, po, 28 days	Moringa Group: *n* = 15; Placebo Group: *n* = 15	MPV & NLR	Not available	Not available
[27]	In vitro HFLS cells from 47-year-old male	Arthritic cell line	Ethanolic MO leaf extract, 75 mg/mL, 24 h	Control RA Cells: *n* = 2; MO Treatment Cells: *n* = 2	35 pathological proteins (NF-κB, NEDD8, LPPRC, S10A6, etc,)	Proteins such as KAIN, HSP71, PDC61, HBA & AK1C1	Not available

Abbreviations: ALP: alkaline phosphatase; ALT: alanine aminotransferase; APRs: acute-phase reactants; AST: aspartate aminotransferase; BDI-II: Beck Depression Inventory-II; BMI: body mass index; CAT: Catalase; CFA: complete Freund’s adjuvant; COX-2: cyclooxygenase-2; CRP: C-reactive protein; DAS28-hsCRP: Disease Activity Score-28 with high-sensitivity C-reactive protein; ESR: erythrocyte sedimentation rate; GSH: glutathione (reduced); Hb: haemoglobin; HDL: high-density lipoprotein; HFLS: human fibroblast-like synoviocyte; hs-CRP: high-sensitivity C-reactive protein; HSP71: heat shock protein 71; I-κB: inhibitor of nuclear factor kappa B; IL: interleukin (e.g., IL-1β, IL-6, IL-10); KAIN: kallistatin; LDL: low-density lipoprotein; LPPRC: leucine-rich PPR motif-containing protein; MDA: malondialdehyde; MO: *Moringa oleifera*; MPV: mean platelet volume; NEDD8: neural precursor cell-expressed, developmentally down-regulated protein 8; NF-κB: nuclear factor kappa-light-chain-enhancer of activated B cells; NLR: neutrophil-to-lymphocyte ratio; PCV: packed cell volume; PDC61: programmed cell death 6-interacting protein; PGE2: prostaglandin E2; po: by mouth; RA: rheumatoid arthritis; RBCs: red blood cells; RF: rheumatoid factor; S10A6: S100 calcium-binding protein A6; SAA: serum amyloid A; SDAI: Simplified Disease Activity Index; SOD: superoxide dismutase; TG: triglycerides; TLC: total leukocyte count; TNF-α: tumour necrosis factor-alpha; VLDL: very low-density lipoprotein; WBCs: white blood cells.

## Data Availability

Not applicable.
